# Does outdoor advertising correlate with retail food purchases made by adolescents? A cross-sectional study in Canada

**DOI:** 10.1093/heapro/daaf016

**Published:** 2025-03-18

**Authors:** Alexander Wray, Gina Martin, Jamie A Seabrook, Sean Doherty, Jason Gilliland

**Affiliations:** Department of Geography & Environment, Western University, 1151 Richmond Street, London, Ontario N6A 3K7, Canada; Human Environments Analysis Lab, Western University, 1151 Richmond Street, London, Ontario N6A 3K7, Canada; Human Environments Analysis Lab, Western University, 1151 Richmond Street, London, Ontario N6A 3K7, Canada; Faculty of Health Disciplines, Athabasca University, 1 University Drive, Athabasca, Alberta T9S 3A3, Canada; Human Environments Analysis Lab, Western University, 1151 Richmond Street, London, Ontario N6A 3K7, Canada; Department of Paediatrics, Brescia School of Food and Nutritional Sciences, Western University, 1151 Richmond Street, London, Ontario N6A 3K7, Canada; Department of Epidemiology & Biostatistics, Western University, 1151 Richmond Street, London, Ontario N6A 3K7, Canada; Human Environments Analysis Lab, Western University, 1151 Richmond Street, London, Ontario N6A 3K7, Canada; Department of Geography & Environmental Studies, Wilfrid Laurier University, 75 University Avenue West, Waterloo, Ontario N2L 3C5, Canada; Department of Geography & Environment, Western University, 1151 Richmond Street, London, Ontario N6A 3K7, Canada; Human Environments Analysis Lab, Western University, 1151 Richmond Street, London, Ontario N6A 3K7, Canada; Department of Epidemiology & Biostatistics, Western University, 1151 Richmond Street, London, Ontario N6A 3K7, Canada; Department of Paediatrics, School of Health Studies, Western University, 1151 Richmond Street, London, Ontario N6A 3K7, Canada

**Keywords:** adolescents, commercial determinants of health, consumer nutrition environment, food environments, food information environment, outdoor advertising, food marketing

## Abstract

Food marketing plays a substantial role in shaping adolescent diets, having wide-ranging ramifications for health behaviours and outcomes throughout the life course. Yet, there remains a dearth of research about how outdoor advertising as a specific channel of food marketing affects purchasing behaviours. We examine self-reported purchases made at retail food outlets by adolescents as it relates to the availability of outdoor food and beverage advertising around each participant’s home, school, and along the journey to and from school. We also consider the impacts of sociodemographics and consumption attitudes on purchasing, as compared to the geographic availability of outdoor advertising. Data are drawn from a survey completed by 545 adolescents in 2018 across four secondary schools in the Middlesex-London region of Ontario, Canada. The availability of outdoor advertising in the home and school environment is marginally correlated with self-reported purchases made at fast food, table-based, grocery, and variety retail outlets. However, consumption attitudes, cultural background, and gender are significantly correlated with purchases, with substantially larger effect sizes. The overall results were consistent between estimating the availability of outdoor advertising in the immediate area surrounding the home and along the journey to and from school. There is considerable health promotion policy interest in regulating outdoor advertising around child-serving locations. However, scarce health promotion resources would be better allocated to educational programming that addresses the substantial role of consumption attitudes in affecting adolescent purchasing behaviour, as compared to the considerably weaker impact of outdoor food advertising observed in our analysis.

**Contribution to Health Promotion**

- Addressing the effects of food marketing on youth health behaviours remains a critical juncture for health promotion intervention, though there is a dearth of evidence on outdoor advertising.
- Outdoor advertising slightly affects adolescent food purchasing behaviour, however, food consumption attitudes and sociodemographics play a more substantial role.
- Health promotion interventions that address outdoor advertising and the availability of unhealthy food at retail outlets should be culturally relevant to the community of interest.
- Future health promotion interventions in Canada should focus on delivering educational programming that improves consumption-related attitudes or addressing more impactful marketing channels.

## INTRODUCTION

The availability of unhealthy retail food outlets and their advertising is viewed as a substantial social determinant of health with wide-ranging ramifications across the life course (Caspi et al. 2012, Health Canada 2017). Furthermore, diet-related behaviours have been linked to a wide range of chronic conditions including heart disease, liver disease, and multiple cancers (Fitzmaurice et al. 2017, Micha et al. 2017). Among adolescents, dietary behaviours have been linked to physical health, mental well-being, social and cognitive development, and academic performance (Schwingshackl et al. 2018). These behaviours are also associated with gender, ethnicity and class; with girls, racialized and lower-income groups having worse diet-related outcomes (Boehm et al. 2021). The investigation of how environmental exposures and competencies affect adolescent diets is important as the attitudes and behaviours formed during these early years may carry on through later life, thereby having long-term impacts on health and wellbeing. This age group remains the ideal target for health promotion interventions that will have lasting impacts throughout the life course.

The food environment as it relates to consumption can be classified into seven spheres of influence—institutional, community, organizational, consumer, information, psychosocial, and perceptual (Glanz et al. 2005). These spheres all contribute to food and beverage consumption behaviours, habits, and preferences (Caspi et al. 2012, Wilkins et al. 2019). The local consumer nutrition environment, or retail food environment, has been identified as an important spatialized component of adolescent food purchasing and dietary behaviours (He et al. 2012, Sadler et al. 2016, Atanasova et al. 2022, Shaw et al. 2022, Polsky et al. 2023). While there has been considerable research into many areas of the consumer nutrition environment, the information environment as it relates to outdoor advertising remains understudied in comparison to the other six domains.

Several studies have assumed a relationship exists between outdoor advertising and purchasing or other health disparities, yet do not include a purchasing or health outcome measure (Lesser et al. 2013, Godin et al. 2017, Signal et al. 2017, Barquera et al. 2018, Egli et al. 2019, Velazquez et al. 2019, Liu et al. 2020, Kennedy et al. 2023). Only one analysis found a slight reduction in the purchasing of high fat, salt, and sugar products at the household level after the introduction of a targeted outdoor advertising ban on public transit (Yau et al. 2022). However, recent reviews suggest an inconclusive relationship between exposures to outdoor advertising, and subsequent purchasing or health-related outcomes for children and adolescents (Boyland et al. 2022, Finlay et al. 2022). Yet, there remains considerable policy interest in the regulation of unhealthy outdoor food and beverage advertising surrounding child-serving locations, such as schools, parks, community centres, and daycares (Hillier et al. 2009, Health Canada 2017, Velazquez et al. 2019, World Health Organization 2021, Sing et al. 2023) even in the absence of evidence that links this specific kind of exposure to behavioural or health outcomes. In Canada, there are no regulations that substantially impact outdoor advertising as a marketing channel for food and beverage products (Health Canada 2017). Given the scarce human, financial, and legislative resources available for health promotion in Canada (Jacques et al. 2023), it is important that recommended policy interventions are rooted in strong evidence that establishes associations between the exposure of interest, in our study’s case: outdoor advertising, with the health outcome or behaviour of interest, in our study’s case: purchasing.

Our study examines adolescents’ purchasing habits at retail food outlets in relation to the spatial *availability* of outdoor food and beverage advertising in the environments surrounding their home and school, as well as along the approximated journey between home and school; while also understanding the *influence* of age, ethnicity, gender, and consumption-based attitudes on purchasing behaviours.

## METHODS

References to outdoor advertising in our analysis encompass both traditional outdoor advertising on billboards, bus shelters, or street-level posters, as well as advertising at the sites of retail food outlets, as their design and placement at these locations are typically advertisement in their own right (Treu 2012, Ozelkan 2019). For our study, outdoor advertisements including those located at retail food outlets are categorized into four types: fast food, table-based, grocery, and variety—following from previously described methods (Tucker et al. 2008). Fast food includes quick-service restaurants, coffee shops, food courts, and other outlets where food is ordered from a counter. This category also includes outlets with limited to no seating that are focused on takeout or delivery. Table-based includes sit-down restaurants, bars, pubs, and other establishments that offer table-based service. Grocery includes stores that sell fresh and packaged food, as well as farmers markets, bakeries, and other more specialized food and beverage retail stores. Variety includes convenience and mart-type outlets that are typically found on residential street corners or in commercial plazas and gas stations selling pre-packaged food and beverages, with little to no fresh groceries available. Advertisements that were related to these food outlets, or products sold at these locations, were assigned to these categories.

### Data collection

Participant data is from a large multi-year intervention study of adolescents attending secondary schools in the census metropolitan area (CMA) of Middlesex-London, a mid-size Canadian region located equidistant between Detroit, Michigan and Toronto, Ontario. Briefly, the SmartAPPetite study from 2015 to 2019 aimed to recruit secondary school-aged children, approximately 13–19 years old, via emails sent to students and parents, in-school posters, and in-school presentations. Students were allocated by school into the intervention or control arm of the study. The intervention consists of a smartphone application that delivers time and location-relevant messaging designed to nudge students towards healthier dietary behaviours (Gilliland et al. 2015). Our analysis only includes baseline survey responses from both intervention and control participants collected during the 2018–19 sampling campaign of the SmartAPPetite study for all participants who reported a valid postal code in the study region (*n* = 545). Surveys were conducted in four secondary schools with provided tablets or the participant’s own device (i.e. wi-fi enabled smartphone, tablet, or laptop). Given the survey took place at baseline, participants were not yet exposed to the intervention. Questions on the baseline survey covered health behaviours, dietary knowledge, consumption attitudes, and purchasing patterns. This study was conducted according to the approved procedures of Western University’s Non-Medical Research Ethics Board (REB #107034) and the Research Assessment Office of the London District Catholic School Board. Written informed assent was obtained from all adolescent participants and written informed consent from their parents.

Data on all retail food outlets in the region were sourced from the local health unit’s food safety inspection database (Middlesex-London Health Unit 2018). The retail food outlet data included the complete addresses of every outlet by type (e.g. fast food, table-based, grocery, and variety). Advertising data was collected through field audits by the study team during the summer of 2018. Photographs were taken of each outdoor billboard, transit shelter, and street-level poster in the entire study region. These photographs were then coded by independent reviewers to determine the food and beverage content of each advertisement (Bowman et al. 2019). It is important to stress that advertisements are often co-located with retail food outlet locations in the Canadian context. Therefore, while we position our analysis as measuring the food information environment, it also is proximally measuring the retail food environment. Given the small number of standalone billboard and transit shelter advertisements in the study region, standalone (*n* = 97) and ancillary (*n* = 1459) outdoor advertising were combined into one measure of the food information environment.

### Data processing

**Figure 1.**
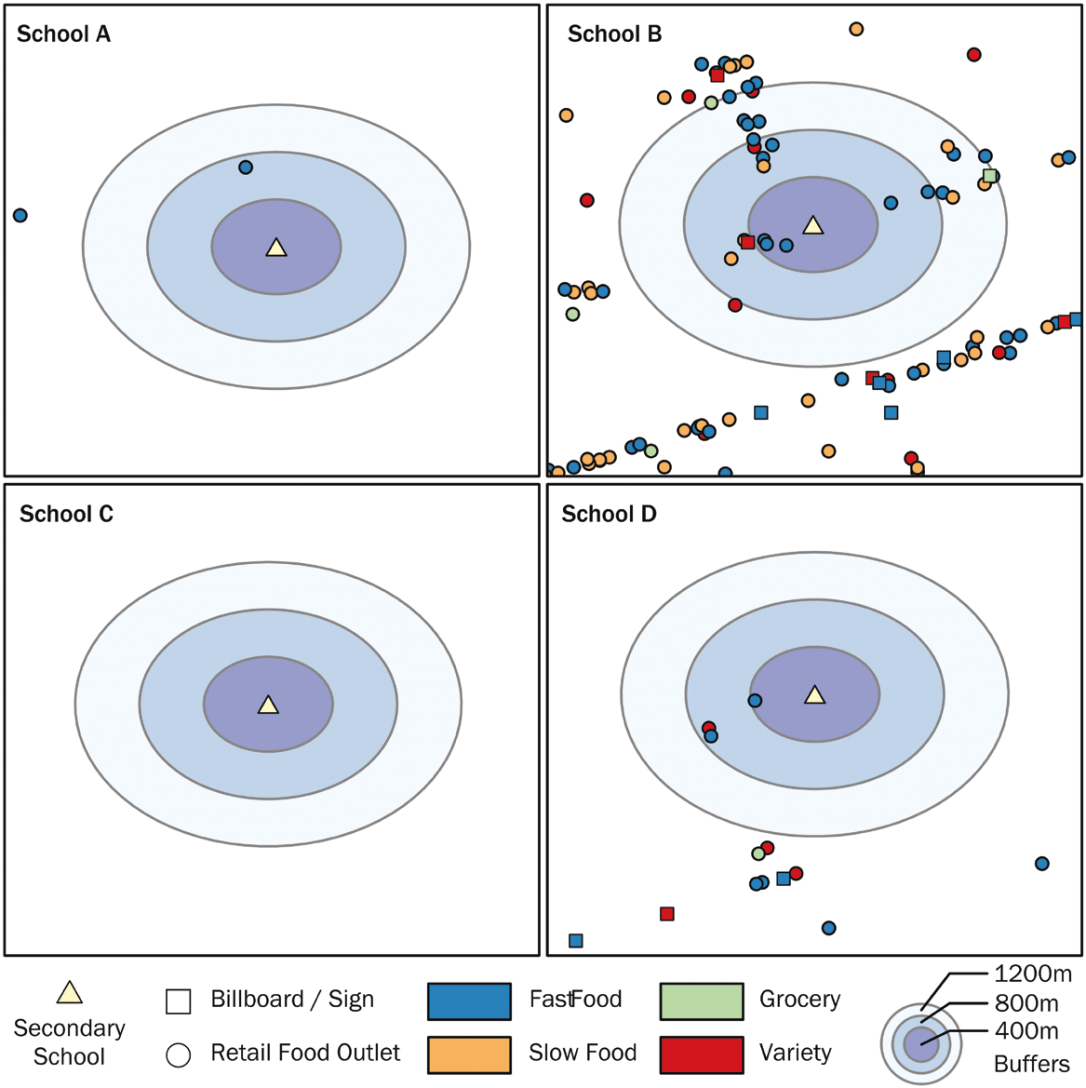
Food information environment surrounding each school. Cartography by Alexander Wray.

For the outcome measure, participants self-reported the number of purchases by retail food outlet type they made on average per week or month, which was then converted so that all measures were a per-month average. Participants were asked to recall any purchases they had made independently. Individual-level variables include age (13–19 years old), self-declared gender identity (boy, girl, or non-binary), and self-declared ethnicity (Asian, Black, Latinx, Middle Eastern, White, and other non-White groups) collected from the survey. Statements about consumption attitudes were coded on a Likert scale (1–5) from strongly disagree (1) to strongly agree (5). Given the large proportion of missing data for household income from individual respondents, an area-level indicator for socioeconomic status was calculated as the percentage of households in the participant’s home postal code that fall below the median household income of the CMA (Statistics Canada 2019). Three of the four schools (Fig. 1) in the analysis have a similar food information environment (<3 ads within 1200 m), so this variable was transformed into a binary variable of the one school with a saturated food information environment (1) and grouping together the three other schools with a sparse food information environment (0).

An 800-m Euclidean buffer is estimated around the centroid of each participant’s postal code to count the number of retail food outlets and advertisements that were available within 800 m of a participant’s home. Furthermore, the shortest network path is estimated between the centroid of the participant’s home postal code and the street entrance of their school using an open-source road and major pathways network dataset. A buffer of 100 m around this shortest network path is used to simulate the visual availability of outdoor advertising along the assumed journey between home and school (Chmielewski and Tompalski 2017).

### Statistical analysis

Poisson models are typically used with dependent count variables; however, food purchases were over-dispersed in our data; therefore, we elected to use a negative binomial regression model for the analysis (Cameron and Trivedi 2013). This choice of test is appropriate given the dependent variable of interest being count-based continuous data, and the theoretical construct being a dose-response relationship between the dependent variable (i.e. purchases) and the independent variable (i.e. advertising) of interest (Venables and Ripley 2002, Cameron and Trivedi 2013). The number of variables included in our models is less than a tenth of the sample size, resulting in appropriate statistical power (Schaafsma et al. 2021). All statistical analyses were completed using R Stats. Negative binomial regression models were run using the *glm.nb* function in the *MASS* package (Venables and Ripley 2002). A variable inflation factor analysis using the *vif* function in the *CAR* package was run to determine if there were any strong correlations between explanatory variables in all models (Fox and Weisberg 2019). In addition, Akaike Information Criterion (AIC) scores were used to compare the overall fit of the models (Burnham and Anderson 2004). Statistical significance is determined at level of *P* < .05. The strength of correlations is defined as very weak: < ± 0.2, weak: ± 0.2–0.4, moderate: ± 0.4–0.6, and strong: < ± 0.6 (Armitage et al. 2002).

For each purchasing outcome (i.e., fast food, table-based, grocery, and variety), which is treated as a continuous variable, a series of models are presented to illustrate differences in assessing advertising availability surrounding the home, or along the journey between home and school. The first group of models examine outdoor advertising availability and sociodemographic variables. Another group of models are fitted with only the sociodemographic and participants’ consumption attitudes. Finally, these two models are combined into one group of models to measure both sets of factors.

## RESULTS

Our analysis included 545 participants attending four different secondary schools collected during the 2018–19 sampling campaign of the SmartAPPetite intervention study (see Supplementary File). Participants ranged in age from 13 to 19 years old, typical of most Ontario, Canada secondary schools. The sample included more girls (*n* = 342) than boys (*n* = 201). There were no participants that identified their gender as non-binary although this was provided as an option on the survey. There is a diverse group of ethnicities in the sample, including Asian (*n* = 65), Black (*n* = 40), Latinx (*n* = 44), Middle Eastern (*n* = 39), White (*n* = 311), and other non-white (*n* = 46) groups. School B (*n* = 180 participants) had a saturated food information environment, while schools A, C, and D (*n* = 365 participants) had a sparse food information environment. All four schools are similar in their sociodemographic composition, being located in suburban settings that serve surrounding households with similar incomes and ethnicities that are reflective of the distributions found in the broader Middlesex-London region.

Outdoor advertising within 800 m of a participant’s home tends to be made up of fast food and variety types. This pattern of fast food (increases to 80% more advertisements) and variety (increases to 76% more advertisements) ad presence is further pronounced when assessing the journey between home and school. Additionally, table-based ads are more present on the journey to and from school as compared to around the home. Grocery advertising is minimal in both contexts.

Participants reported a range of consumption attitudes towards food along a Likert scale, from strongly disagree (1) to strongly agree (5). Most agreed that *eating healthy food is important to me* (x̄: 3.16 ± 0.67) and *preparing lunch at home saves money* (x̄: 3.26 ± 0.74); however, some expressed *preparing lunch at home takes too much time* (x̄: 2.31 ± 1.11). In addition, some expressed that *I like to cook* (x̄: 2.74 ± 0.93), that *cooking meals helps me eat healthier* (x̄: 2.74 ± 0.91), and many expressed that *I have no problem understanding food labels* (x̄: 2.96 ± 0.87).

### Relationship between purchasing and advertising availability

Fast food ads around the home have a positive significant correlation while variety ads have a significant negative correlation with fast food purchases (Table 1). These correlations are not found when examining the journey to and from school. Participants attending the school with a saturated food information environment make more fast-food purchases than their peers at other schools. Boys made significantly less table-based purchases than girls, while Latinx participants had a significantly higher count of table-based purchases in both models. The school food information environment does not significantly relate to table-based purchases.

**Table 1.** Model summary for fast food and table-based purchases with advertising availability factors

	Fast food purchases [IRR (95% CI)]		Table-based purchases [IRR (95% CI)]	
	*Home*	*Journey*	*Home*	*Journey*
Fast food ads	**1.02 (1.00, 1.05)**	0.99 (0.97, 1.01)	**1.04 (1.01, 1.07)**	**0.98 (0.95, 1.00)**
Slow food ads	0.98 (0.94, 1.01)	1.01 (0.98, 1.04)	0.98 (0.94, 1.02)	1.01 (0.98, 1.04)
Grocery ads	0.99 (0.89, 1.10)	1.05 (0.97, 1.14)	0.96 (0.84, 1.10)	1.01 (0.91, 1.12)
Variety ads	**0.94 (0.90, 0.99)**	1.00 (0.97, 1.04)	**0.90 (0.85, 0.96)**	1.01 (0.98, 1.05)
Gender, boys	0.89 (0.76, 1.04)	0.88 (0.75, 1.03)	**0.79 (0.66, 0.96)**	**0.81 (0.67, 0.98)**
Age	1.01 (0.95, 1.08)	1.02 (0.96, 1.08)	0.97 (0.90, 1.05)	0.98 (0.91, 1.06)
*Ethnicity (ref. White)*				
Asian	0.86 (0.67, 1.10)	0.87 (0.68, 1.12)	1.07 (0.80, 1.43)	1.06 (0.79, 1.42)
Black	1.16 (0.86, 1.55)	1.15 (0.85, 1.54)	0.78 (0.53, 1.16)	0.77 (0.52, 1.42)
Latinx	0.92 (0.68, 1.25)	0.88 (0.65, 1.19)	**1.79 (1.31, 2.45)**	**1.72 (1.25, 2.37)**
Middle Eastern	0.84 (0.61, 1.15)	0.89 (0.65, 1.21)	0.79 (0.54, 1.17)	0.82 (0.56, 1.20)
Other non-white	1.15 (0.87, 1.51)	1.13 (0.86, 1.49)	0.89 (0.63, 1.25)	0.86 (0.61, 1.22)
% Below CMA income	1.61 (0.96, 2.69)	1.24 (0.75, 2.03)	1.43 (0.77, 2.65)	1.19 (0.66, 2.14)
Saturated school	**1.26 (1.06, 1.51)**	**1.40 (1.12, 1.75)**	1.01 (0.82, 1.26)	1.14 (0.87, 1.49)
Observations	494	494	487	487
Cragg–Uhler *R*^2^	0.06	0.05	0.07	0.07
Akaike inf. crit.	2,674.31	2,680.18	1,703.73	1,706.30

*Note:* Incidence rate ratio (IRR), 95% confidence interval (CI), *P* < .05 values are bolded.

Grocery and variety purchases appear to be influenced by several factors (Table 2). Table-based ads have a negative correlation with grocery purchases in both home and journey-based settings, while fast food ads have a marginal positive correlation along the journey to and from school. Boys have a 13% lower rate than girls in making grocery purchases, and those with a Middle Eastern background make more grocery purchases as compared to other ethnicities. Although the model of the home environment shows that participants from areas with a high number of households below the median household income in the region are significantly less likely to make a grocery purchase, this correlation is not found when modelling the journey to and from school. Variety purchases appear to be primarily influenced by ethnicity. Blacks, Latinx, and other non-white participants are more likely to make a variety purchase. Participants in the saturated school food environment are also more likely to make more variety purchases than their peers in less saturated environments.

**Table 2.** Model summary for grocery and variety purchases with advertising availability factors

	Grocery purchases [IRR (95% CI)]		Variety purchases [IRR (95% CI)]	
	*Home*	*Journey*	*Home*	*Journey*
Fast food ads	1.01 (0.99, 1.03)	**1.02 (1.00, 1.04)**	1.02 (0.99, 1.05)	1.00 (0.98, 1.03)
Slow food ads	**0.98 (0.95, 1.00)**	**0.97 (0.95, 0.99)**	0.98 (0.93, 1.02)	1.00 (0.97, 1.03)
Grocery ads	0.97 (0.89, 1.06)	0.95 (0.89, 1.02)	0.97 (0.85, 1.11)	0.98 (0.89, 1.09)
Variety ads	1.02 (0.98, 1.06)	1.01 (0.99, 1.04)	0.97 (0.91, 1.03)	1.00 (0.96, 1.04)
Gender, boys	**0.87 (0.77, 1.00)**	**0.86 (0.76, 0.98)**	1.10 (0.90, 1.35)	1.11 (0.91, 1.35)
Age	0.98 (0.93, 1.03)	0.98 (0.93, 1.03)	0.98 (0.91, 1.06)	0.98 (0.91, 1.06)
*Ethnicity (ref. White)*				
Asian	1.04 (0.84, 1.27)	1.04 (0.85, 1.28)	1.30 (0.95, 1.77)	1.33 (0.98, 1.82)
Black	0.98 (0.76, 1.25)	0.94 (0.74, 1.21)	**1.51 (1.04, 2.20)**	**1.47 (1.01, 2.13)**
Latinx	1.08 (0.84, 1.39)	1.06 (0.82, 1.36)	**1.57 (1.08, 2.29)**	**1.49 (1.02, 2.19)**
Middle Eastern	**1.28 (1.00, 1.63)**	**1.30 (1.02, 1.65)**	1.18 (0.79, 1.74)	1.20 (0.82, 1.77)
Other non-white	0.87 (0.69, 1.10)	0.83 (0.66, 1.05)	**1.56 (1.11, 2.19)**	**1.54 (1.10, 2.17)**
% Below CMA income	**0.59 (0.39, 0.90)**	0.82 (0.55, 1.22)	0.86 (0.45, 1.65)	0.84 (0.45, 1.58)
Saturated school	1.00 (0.86, 1.16)	0.86 (0.71, 1.03)	**1.35 (1.07, 1.69)**	1.31 (0.99, 1.73)
Observations	493	493	487	487
Cragg–Uhler *R*^2^	0.05	0.06	0.05	0.04
Akaike inf. crit.	2,581.89	2,576.43	2,284.98	2,287.09

*Note:* Incidence rate ratio (IRR), 95% confidence interval (CI), *P* < .05 values are bolded.

### Relationship between purchases and consumption attitudes

The second group of models combines individual and environment variables with measures of consumption attitudes (Table 3). Participants who express that eating healthy food is important to them are significantly less likely to purchase fast food. Participants who expressed they were confident with food labels are more likely to make a table-based or grocery purchase. Those who like to cook are less likely to make fast food and table-based purchases. Those who prepare their lunch to save money are less likely to make fast food purchases while those who said they did not have enough time to prepare their lunch for school are more likely to make fast food and variety purchases, and less likely to make grocery purchases. School and ethnicity had similar relationships to the previous set of models across all types of purchases.

**Table 3.** Model summary for consumption attitudes and purchases

	Type of purchases [IRR (95% CI)]			
	*Fast food*	*Table-based*	*Grocery*	*Variety*
Healthy eating	**0.80 (0.65, 0.93)**	0.90 (0.73, 1.05)	1.02 (0.91, 1.11)	1.00 (0.83, 1.14)
Know labels	1.03 (0.94, 1.11)	**1.11 (1.01, 1.20)**	**1.07 (1.00, 1.14)**	1.08 (0.97, 1.18)
Like to cook	0.90 (0.80, 0.99)	**0.85 (0.72, 0.96)**	1.01 (0.93, 1.08)	0.96 (0.83, 1.07)
Cook for health	1.02 (0.93, 1.11)	1.09 (0.99, 1.19)	0.94 (0.86, 1.02)	0.96 (0.83, 1.07)
Cook to save	**0.87 (0.75, 0.98)**	0.99 (0.85, 1.11)	0.95 (0.85, 1.04)	0.88 (0.72, 1.02)
No time to cook	**1.12 (1.06, 1.18)**	1.06 (0.98, 1.13)	**0.94 (0.87, 1.00)**	**1.11 (1.02, 1.18)**
Gender, boys	0.89 (0.76, 1.04)	**0.77 (0.63, 0.94)**	**0.83 (0.73, 0.95)**	1.08 (0.88, 1.32)
Age	1.00 (0.94, 1.06)	0.95 (0.88, 1.02)	0.99 (0.94, 1.05)	0.97 (0.90, 1.05)
*Ethnicity (ref. White)*				
Asian	0.87 (0.68, 1.12)	1.11 (0.83, 1.49)	1.02 (0.83, 1.26)	1.30 (0.95, 1.77)
Black	1.17 (0.87, 1.56)	0.72 (0.48, 1.09)	0.98 (0.76, 1.26)	**1.53 (1.05, 2.23)**
Latinx	0.88 (0.65, 1.18)	**1.66 (1.21, 2.26)**	1.09 (0.85, 1.40)	**1.53 (1.05, 2.22)**
Middle Eastern	0.91 (0.67, 1.23)	0.84 (0.58, 1.22)	**1.34 (1.06, 1.70)**	1.22 (0.83, 1.78)
Other non-white	1.16 (0.89, 1.51)	0.87 (0.62, 1.23)	0.86 (0.68, 1.10)	**1.53 (1.09, 2.15)**
% Below CMA income	1.18 (0.74, 1.87)	1.11 (0.63, 1.95)	0.70 (0.47, 1.03)	0.80 (0.44, 1.46)
Saturated school	**1.23 (1.04, 1.46)**	0.98 (0.80, 1.21)	1.03 (0.89, 1.20)	**1.36 (1.09, 1.69)**
Observations	489	482	488	482
Cragg–Uhler *R*^2^	0.12	0.08	0.06	0.07
Akaike inf. crit.	2,623.13	1,688.35	2,558.67	2,259.56

*Note:* Incidence rate ratio (IRR), 95% confidence interval (CI), *P* < .05 values are bolded.

### Relationships between purchases, advertising availability, and consumption attitudes

Fast food and table-based purchases are more highly correlated with consumption attitudes than the availability of outdoor advertising (Table 4). The availability of variety ads has a negative correlation with fast food purchasing, while those who placed importance on healthy eating are significantly less likely to make a fast food purchase. Those who expressed an affinity for cooking and packed their lunch to save money are less likely to make a fast food purchase, while those who said they did not have enough time to pack lunch are significantly more likely to purchase fast food. Participants who attend the school with a saturated retail food environment are more likely to purchase fast food than their peers in sparse school food environments. Participants who like to cook are significantly less likely to make a table-based purchase. Boys are significantly less likely than girls to make a table-based purchase, while Latinx participants are significantly more likely to purchase from a table-based outlet.

**Table 4.** Model summary for fast food and table-based purchases with all factors

	Fast food purchases [IRR (95% CI)]		Table-based purchases [IRR (95% CI)]	
	*Home*	*Journey*	*Home*	*Journey*
Fast food ads	1.02 (0.99, 1.04)	1.00 (0.98,1.02)	**1.03 (1.00, 1.07)**	0.98 (0.95, 1.00)
Slow food ads	0.99 (0.95, 1.02)	0.99 (0.97,1.02)	0.99 (0.95, 1.02)	1.01 (0.97, 1.04)
Grocery ads	0.97 (0.87, 1.08)	1.02 (0.94,1.10)	0.95 (0.84, 1.08)	1.00 (0.90, 1.11)
Variety ads	**0.94 (0.90, 0.99)**	1.00 (0.97,1.03)	**0.91 (0.85, 0.97)**	1.02 (0.98, 1.06)
Healthy eating	**0.78 (0.63, 0.92)**	**0.79 (0.64,0.93)**	0.88 (0.70, 1.03)	0.91 (0.75, 1.06)
Know labels	1.01 (0.93, 1.10)	1.03 (0.94,1.11)	1.09 (0.99, 1.19)	**1.12 (1.02, 1.21)**
Like to cook	**0.91 (0.91, 1.00)**	**0.90 (0.81,0.99)**	**0.86 (0.74, 0.97)**	**0.86 (0.74, 0.97)**
Cook for health	1.01 (0.92, 1.10)	1.02 (0.93,1.11)	1.09 (0.98, 1.18)	1.09 (0.98, 1.18)
Cook to save	**0.88 (0.76, 0.99)**	**0.88 (0.75,0.99)**	1.00 (0.86, 1.12)	0.98 (0.84, 1.10)
No time to cook	**1.12 (1.05, 1.18)**	**1.12 (1.06,1.18)**	1.05 (0.96, 1.12)	1.05 (0.97, 1.13)
Gender, boys	0.90 (0.77, 1.05)	0.89 (0.76,1.04)	**0.78 (0.64, 0.95)**	**0.79 (0.65, 0.96)**
Age	0.99 (0.93, 1.06)	0.99 (0.93,1.06)	0.94 (0.88, 1.02)	0.96 (0.89, 1.03)
*Ethnicity (ref. White)*				
Asian	0.86 (0.68, 1.11)	0.88 (0.69, 1.13)	1.09 (0.82, 1.46)	1.07 (0.80, 1.44)
Black	1.18 (0.89, 1.57)	1.17 (0.87, 1.56)	0.74 (0.49, 1.11)	0.73 (0.48, 1.09)
Latinx	0.92 (0.68, 1.24)	0.89 (0.66, 1.20)	**1.80 (1.31, 2.46)**	**1.72 (1.25, 2.36)**
Middle Eastern	0.92 (0.68, 1.25)	0.92 (0.68, 1.25)	0.84 (0.57, 1.23)	0.84 (0.62, 1.23)
Other non-white	1.17 (0.90, 1.53)	1.15 (0.88, 1.50)	0.89 (0.63, 1.25)	0.87 (0.62, 1.23)
% Below CMA income	1.51 (0.91, 2.49)	1.16 (0.72, 1.87)	1.38 (0.75, 2.55)	1.16 (0.65, 2.08)
Saturated school	**1.23 (1.03, 1.46)**	**1.25 (1.00, 1.55)**	0.99 (0.80, 1.22)	1.09 (0.83, 1.43)
Observations	489	489	482	482
Cragg–Uhler *R*^2^	0.13	0.12	0.10	0.10
Akaike inf. crit.	2,622.81	2,630.55	1,685.37	1,687.43

*Note:* Incidence rate ratio (IRR), 95% confidence interval (CI), *P* < .05 values are bolded.

Grocery and variety store purchases are more correlated with sociodemographic factors than consumption attitudes and advertising availability (Table 5). Table-based ads have a negative correlation with grocery purchasing. Participants who are confident with food labels are more likely to make a grocery purchase, and those who do not have enough time to pack lunch are less likely to make a grocery purchase. Boys are less likely to make a grocery purchase than girls. Variety store purchases are more affected by ethnicity with Black, Latinx, and other non-white populations more likely to make a purchase. Those who do not have time to pack their lunch are more likely to make a variety purchase.

**Table 5.** Model summary for grocery and variety purchases with all factors

	Grocery purchases [IRR (95% CI)]		Variety purchases [IRR (95% CI)]	
	*Home*	*Journey*	*Home*	*Journey*
Fast food ads	1.01 (0.99, 1.03)	1.01 (1.00, 1.03)	1.02 (0.99, 1.05)	1.01 (0.98, 1.04)
Slow food ads	**0.98 (0.95, 1.00)**	**0.97 (0.95, 0.99)**	0.98 (0.94, 1.02)	0.99 (0.96, 1.02)
Grocery ads	0.97 (0.89, 1.06)	0.95 (0.88, 1.01)	0.96 (0.84, 1.10)	0.98 (0.88, 1.09)
Variety ads	1.02 (0.98, 1.06)	1.02 (0.99, 1.04)	0.98 (0.92, 1.04)	1.00 (0.96, 1.04)
Healthy eating	1.02 (0.92, 1.11)	1.00 (0.89, 1.09)	0.99 (0.82, 1.13)	0.99 (0.82, 1.13)
Know labels	**1.07 (1.00, 1.14)**	**1.08 (1.01, 1.14)**	1.08 (0.97, 1.18)	1.08 (0.97, 1.18)
Like to cook	1.01 (0.93, 1.08)	1.00 (0.93, 1.07)	0.96 (0.84, 1.07)	0.95 (0.83, 1.07)
Cook for health	0.95 (0.87, 1.03)	0.95 (0.86, 1.02)	0.95 (0.82, 1.07)	0.96 (0.83, 1.07)
Cook to save	0.95 (0.86, 1.04)	0.94 (0.85, 1.03)	0.89 (0.73, 1.03)	0.88 (0.71, 1.02)
No time to cook	**0.94 (0.87, 1.00)**	**0.94 (0.87, 1.00)**	**1.10 (1.02, 1.18)**	**1.11 (1.02, 1.18)**
Gender, boys	**0.84 (0.73, 0.96)**	**0.82 (0.72, 0.94)**	1.08 (0.88, 1.32)	1.08 (0.88, 1.32)
Age	0.99 (0.94, 1.04)	0.99 (0.94, 1.04)	0.97 (0.90, 1.05)	0.97 (0.89, 1.05)
*Ethnicity (ref. White)*				
Asian	1.02 (0.83, 1.26)	1.03 (0.84, 1.27)	1.28 (0.93, 1.75)	1.31 (0.96, 1.80)
Black	0.98 (0.76, 1.27)	0.95 (0.74, 1.22)	**1.55 (1.07, 2.27)**	**1.50 (1.02, 2.19)**
Latinx	1.10 (0.85, 1.41)	1.07 (0.83, 1.37)	**1.59 (1.09, 2.32)**	**1.53 (1.04, 2.25)**
Middle Eastern	1.26 (0.99, 1.61)	**1.27 (1.00, 1.61)**	1.20 (0.81, 1.78)	1.22 (0.82, 1.79)
Other non-white	0.88 (0.70, 1.12)	0.85 (0.67, 1.07)	**1.55 (1.10, 2.18)**	**1.53 (1.08, 2.15)**
% Below CMA income	**0.60 (0.39, 0.92)**	0.82 (0.55, 1.22)	0.85 (0.44, 1.63)	0.85 (0.46, 1.59)
Saturated school	1.02 (0.87, 1.18)	0.88 (0.73, 1.06)	**1.34 (1.07, 1.68)**	1.26 (0.95, 1.67)
Observations	488	488	482	482
Cragg–Uhler *R*^2^	0.07	0.08	0.07	0.07
Akaike inf. crit.	2,560.76	2,554.08	2,266.01	2,266.81

*Note:* Incidence rate ratio (IRR), 95% confidence interval (CI), *P* < .05 values are bolded.

Variance inflation factor analyses revealed no multicollinearity issues in any of the models. Due to missingness, some participants were excluded from the fitted models.

## DISCUSSION

The availability of retail food advertisements is weakly correlated with fast food, table-based, grocery, and variety purchasing when examining effect sizes and overall model diagnostics. However, a participant’s consumption attitudes appear to have a stronger, albeit still weak correlation, in addition to the moderate correlations found with ethnicity and gender across all types of purchases. Among adolescents, fast food and variety purchases may be more frequent than table-based purchases given the higher cost of table-based outlets, and the supportiveness of fast food outlets for socialization among this age group (Neufeld et al. 2022). This difference creates potential health implications, as in the Canadian context table-based outlets may have a greater variety of healthy options than fast food outlets and the pre-packaged options available from variety outlets.

Food and beverage purchasing among adolescents is affected by a range of individual and environment-based factors. Gender and ethnicity, coupled with individual attitudes about eating and cooking were found to relate significantly to food purchasing. The availability of advertising in the home neighbourhood environment, along with the availability of advertising on the journey between home and school had weaker correlations with table-based and grocery purchasing than compared to those same correlations when examining consumption attitudes. Additionally, the food information environment surrounding school correlates positively with adolescents’ fast food and variety purchasing patterns. Previous studies have found that the food retail environment around schools may impact consumption patterns among adolescents (Hillier et al. 2009, He et al. 2012, Barquera et al. 2018, Egli et al. 2019, Velazquez et al. 2019). Our study also establishes a weak correlation between the food information environment and purchasing. Students attending the school with a saturated food information environment have a level of purchasing 1.23–1.34 times greater than students attending a school with a sparse food information environment. In particular, the high availability of fast food and variety advertising, and the availability of retail food outlets associated with that advertising, around schools may be overly influencing adolescents’ consumption patterns in comparison to their peers.

Consumption attitudes also correlate significantly with purchasing patterns for many adolescents. The correlations between particular attitudes and types of purchases observed in our analysis align with prior research (Kinard and Webster 2012, Stok et al. 2016). Gender and ethnicity-based differences found in our analysis align with other studies that found boys make fewer purchases than girls, and sociocultural factors influence purchasing behaviours (Yancey et al. 2009, He et al. 2012, Bragg et al. 2017, Godin et al. 2017). Overall, boys are less likely than girls to make table-based and grocery purchases, which may be the result of gender-based norms around food and shopping (Flagg et al. 2014). Similarly, the correlation based on ethnicity could be the result of cultural norms in some groups around the types of retail food outlets that are frequented, or the clustering of non-white participants in particular areas that may result in a proxy effect for their local consumer nutrition environment, likely because cultural communities self-select into neighbourhoods with culturally relevant outlets and amenities (Mendez et al. 2006).

While we observed differences in purchasing by household income measured at a neighbourhood scale, with participants living in lower-income neighbourhoods being less likely to make grocery purchases, we are cautious to interpret these findings. First, prior work has found no socioeconomic-based patterning of outdoor advertising (Liu et al. 2022, Robertson et al. 2022). Second, the substitution of an areal measure for an individual self-reported one may create a proxy measure of other factors related to the overall socioeconomic attractiveness of a neighbourhood, such as the presence of a food desert or income-based deprivation (Larsen and Gilliland 2008). In the case of grocery stores in North America, many are located in periphery commercial areas along main arterial avenues isolated from most central older and lower-income residential neighbourhoods (Larsen and Gilliland 2008, Treu 2012). However, this finding could be a signal that grocery and variety purchasing is affected by the purchasing power of adolescents, as well as the surrounding land uses that enable some adolescents to have greater geographic accessibility to these types of retail food outlets.

The lack of differences between the models suggests that the spatial construct of measuring potential advertising availability has little influence on the overall fit of the model. However, many explanatory variables with significant correlations in the home-based model were reduced in the approximated journey to and from the school model, likely from the incorporation of less saturated school environments in the advertising availability measure under the journey to and from the school model. Moreover, the proportion of households below the median household income in the CMA had a significant negative correlation with grocery purchases in the buffer model. However, the journey to and from school model showed a weaker correlation. Many of the correlations between purchasing and advertising availability were reduced from the home-based model to the journey-based model. This difference in the strength of correlations demonstrates the importance of considering the geographic scale and the spatial construct being measured in the analysis, particularly as it relates to the food information environment (Wray et al. 2021). Thus, many participants have low availability of outdoor advertising in their immediate home environment, but high availability on the journey between home and school as they have to take major arterial roads with high commercial saturation to reach their school.

### Limitations

There are four limitations to our study. First, the synthesis of data from multiple sources with data collection at slightly different times may result in a spatiotemporal mismatch between the dependent and explanatory variables. The advertising and food environment data were captured from May to October 2018, while the survey data were collected in September 2018. Given the phrasing of the survey question for the dependent variable as a participant’s typical behaviour rather than a recall of the past week of purchases, there may be a temporal mismatch in the data. The impact of this mismatch should be minimal however, as most Canadian outdoor advertising campaigns typically last 1 month with advertisers in the food and beverage industry typically returning to the same locations, usually just altering the product or promotion being advertised on the billboard (Ozelkan 2019).

Second, this study may misclassify some of the spatial relationships due to not using the adolescents’ exact home addresses. Although postal code centroids are the most used address proxy in Canadian environment and health research, the use of postal code centroids to represent the adolescent participants’ home locations may misclassify potential exposures, particularly in rural locations where postal code areas are much larger than urban areas. Nevertheless, previous research has shown postal code centroids to be acceptable address proxies in Canadian urban and suburban areas (Healy and Gilliland 2012) which aligns with the composition of the population included in our study.

Third, the study may be impacted using self-reported measures rather than observed data. Although self-reported measures are more common than observational measures in dietary research, they are known to be affected by biases related to social desirability and performativity. For example, adolescents may want to impress their peers and perform ‘well’ on the survey questions (Hebert et al. 2008). In addition, the cross-sectional nature of our study likely underestimates the influence that cumulative exposure to advertising would have on behaviour. Unfortunately, the collection of observed longitudinal data in relation to purchasing is incredibly challenging with a large sample as it typically involves approaches such as plate photography, direct observation by researchers during mealtimes, or potentially privacy-invasive automated data collection technologies like wearable cameras.

Fourth, the survey did not collect data about the other types of food and beverage advertising that adolescents experience, such as on television, radio, and social media. While this unaccounted factor may explain some of the variances observed in purchasing, they are beyond the remit of this study which exclusively focuses on outdoor advertising channels.

### Implications

We provide considerable improvements over the existing literature which has attempted to link outdoor advertising availability with food and beverage purchasing and other health-related behaviours, without relevant outcome measures. The use of a purchasing measure as the outcome variable in our analysis confirms the assumptions made about outdoor advertising in prior studies and improves upon them by separately analysing fast food, table-based, grocery, and variety purchasing habits. Whilst outdoor advertising, and more likely the retail food environment surrounding schools, in some contexts, appears to weakly influence purchasing; consumption-related attitudes, knowledge, and skills have a more substantial impact on purchasing.

Future research should use more complex spatial analysis techniques to ascertain the effects of true exposure and engagement with outdoor advertising on purchasing rather than potential exposure. Such advances would best be served by using direct spatiotemporal measurements of activities, mobility, and exposure in everyday environments by geospatial data logging (Wray et al. 2021). Given the number of relationships in this study that were statistically significant with weak correlations along with the weak explanatory power of the models, further studies with a larger sample size may enable more definitive conclusions to be drawn about the correlations between outdoor advertising exposure and purchasing. Future research should also ideally account for the other advertising channels that adolescents may be exposed to in their daily lives, particularly those on social media.

Our findings support growing policy and practitioner interest in regulating and addressing the negative health effects of advertising on health behaviours (Health Canada 2017, World Health Organization 2021). Targeting legislative efforts to regulate the food information and retail environment surrounding schools could be an effective approach to reducing unhealthy purchasing behaviour by adolescents. However, our analysis suggests scarce health promotion policy resources could be better allocated towards promoting healthier purchasing behaviours through education and skills development rather than attempting to regulate outdoor advertising, as was proposed by Health Canada (2017). Indeed, attempts to regulate outdoor advertising content or even the locations of retail food outlets may not be achievable or an effective use of public resources in many constitutional democratic systems, given the risks of strong organized opposition from commercial actors (Sing et al. 2023). Instead, the provision of accessible and affordable school food programs, which are not widely available in Canada, could reduce the attractiveness of nearby fast food and variety outlets, whilst also ameliorating ethnic and class-based disparities in food access and affordability (Colley et al. 2019, Ismail et al. 2023). Required courses in secondary schools that focus on meal planning, food preparation, and cooking could promote more independent grocery shopping behaviour among adolescents, whilst simultaneously increasing at-home preparation of food (Ekwaru et al. 2021, Lee et al. 2023, Ares et al. 2024).

### Conclusion

In summary, this research area is continuing to evolve. We stress that this subfield of health promotion research should not rely on generalized advertising research to draw conclusions about the specific effects of outdoor food and beverage advertising on consumption by adolescents. Future work should examine the food information environment within the context of each channel of potential communication (Kelly et al. 2023), as well as consider other avenues for policy intervention that would achieve similar outcomes. In our view, scarce public resources dedicated to health promotion are being mistargeted towards outdoor advertising. Instead, these resources should be used to improve food-related skills and knowledge among adolescents or address more impactful advertising channels.

## Supplementary Material

daaf016_suppl_Supplementary_Material

## Data Availability

None.
