# Supplementary material for: Does outdoor advertising correlate with retail food purchases made by adolescents? A cross-sectional study in Canada
Source: Health Promot Int. 2025 Mar 18;40(2):daaf016. doi: 10.1093/heapro/daaf016 (PMC11915500; doi:10.1093/heapro/daaf016)
Supplement: daaf016_suppl_Supplementary_Material [file daaf016_suppl_supplementary_material.docx]

**SUPPLEMENTARY FILE**

**Table 1.** Summary of individual and environment variables from participants

| **Individual Variables** | | |  | **Environment Variables** | | | |
| --- | --- | --- | --- | --- | --- | --- | --- |
| *Variable* | Category | n (%) |  | *Variable* | | Category | n (%) |
| *Age (years)* | | |  | *% Households below CMA median income (continuous)^1^* | | | |
|  | 13 | 15 (3%) |  |  | | < 20% | 13 (2%) |
|  | 14 | 129 (23%) |  |  | | 20 – 39% | 161 (30%) |
|  | 15 | 132 (24%) |  |  | | 40 – 59% | 183 (34%) |
|  | 16 | 129 (23%) |  |  | | 60 – 79% | 143 (26%) |
|  | 17 | 115 (21%) |  |  | | > 80% | 45 (8%) |
|  | 18 | 17 (4%) |  | *School (binary, School B vs. all other schools)* | | | |
|  | 19 | 2 (1%) |  |  | | School A | 75 (14%) |
|  | Missing | 6 (1%) |  |  | | School B | 180 (33%) |
|  | | |  |  | | School C | 209 (38%) |
| *Gender identity (binary, ref. Girl)* | | |  |  | | School D | 81 (15%) |
|  | Boy | 201 (37%) |  | *Advertising availability (continuous)^1^* | | | |
|  | Girl | 342 (62%) |  | *Type* | *Ads* | *Home (800m)* | *Journey (100m)* |
|  | Missing | 2 (1%) |  | Fast Food | 0 | 166 (30%) | 108 (20%) |
|  |  |  |  |  | >1 | 379 (70%) | 437 (80%) |
| *Ethnicity (categorical, ref. White)* | | |  | Slow Food | 0 | 246 (45%) | 119 (22%) |
|  | Asian | 65 (12%) |  |  | >1 | 299 (55%) | 426 (78%) |
|  | Black | 40 (7%) |  | Grocery | 0 | 353 (65%) | 405 (74%) |
|  | Latinx | 44 (8%) |  |  | > 1 | 192 (35%) | 140 (26%) |
|  | Middle Eastern | 39 (7%) |  | Variety | 0 | 207 (38%) | 132 (24%) |
|  | White | 307 (56%) |  |  | >1 | 338 (62%) | 413 (76%) |
|  | Other | 46 (8%) |  | All types | 0 | 117 (21%) | 87 (16%) |
|  | Missing | 4 (2%) |  |  | >1 | 428 (79%) | 458 (84%) |
| *Note:* | ^1^ *Variables are presented in categorical formats in this table to illustrate distributions among participants. They are treated as continuous or binary variables as noted in the analysis.* | | | | | | |
